# LncRNA-AK149641 associated with airway inflammation in an OVA-induced asthma mouse model

**DOI:** 10.1007/s10863-020-09844-6

**Published:** 2020-09-14

**Authors:** Jie Zhang, Yao Zhou, Haiyan Gu, Jiamin Zhang, Heng Tang, Qiangquan Rong, Lina Gu, Jing Pan, Deyu Zhao, Feng Liu

**Affiliations:** 1 Department of Respiratory Medicine, Children’s Hospital of Nanjing Medical University, Nanjing, 210008 Jiangsu China; 2 Department of Emergency Medicine, Children’s Hospital of Nanjing Medical University, Nanjing, 210008 Jiangsu China; 3 Department of Pediatrics, Gaochun People’s Hospital, Nanjing, 211300 Jiangsu China; 4 Wuxi Children’s Hospital, Wuxi, 214000 Jiangsu China

**Keywords:** lncRNA-AK149641, OVA-induced asthma mouse model, TNF-α, IL-6 and NF-κB signaling pathway

## Abstract

Asthma is defined as a heterogeneous disease, usually characterized by chronic airway inflammation. Long noncoding RNAs (lncRNAs) play important roles in various biological processes. To know more about the relationships between lncRNAs and asthma, gene microarray analysis was performed to screen differentially expressed lncRNAs between the lung tissue of ovalbumin (OVA) mice and control mice. Further studies showed that downregulating differentially expressed lncRNA-AK149641 by adeno-associated virus 6 (AAV6) in OVA mice inhibited airway inflammation, with improved airway compliance and resistance, diminished infiltration of inflammatory cells, as well as less secretions of mucus, tumor necrosis factor alpha (TNF-α) and interleukin-6 (IL-6). Moreover, the activity of nuclear factor-kappa B (NF-κB) in the lung tissue was reduced after downregulating lncRNA-AK149641. In conclusion, we proposed that downregulation of lncRNA-AK149641 attenuated the airway inflammatory response in an OVA-induced asthma mouse model, probably in association with modulation of the NF-κB signaling pathway.

## Introduction

Bronchial asthma are a heterogeneous group of respiratory diseases, which is common among children and whose incidence is increasing in developed nations (Wilson et al. 2006). It is characterized by airway inflammation and airway hyperresponsiveness (AHR) with symptoms including recurrent wheeze, cough, and shortness of breath, together with variable expiratory airflow limitation (Kim et al. 2010). It has been widely accepted that inflammatory cells, such as eosinophils and mast cells (Doherty and Croft 2011; Galli et al. 2011; Kim et al. 2010), as well as various cytokines, including TNF-α and IL-6, take part in the airway inflammatory response of asthma (Doganci et al. 2005; Rameshwar et al. 2014; Rincon and Irvin 2012).

LncRNAs are RNAs with more than 200 nucleotides without a protein-coding capability (Yang et al. 2014). Due to a lack of reading frames, lncRNAs were initially considered non-functional. However, accumulating evidence has suggested that they participate in a variety of biological and pathological processes, such as cancer (Zhou et al. 2015c), self-renewal of embryonic stem cells (Tu et al. 2018), metabolism (Yin et al. 2015), and immune responses (Houtman et al. 2018). Moreover, an increasing number of studies have reported that lncRNAs take part in asthmatic airway inflammation. For example, the lncRNA BCYRN1 promotes the proliferation and migration of asthmatic rat airway smooth muscle cells (ASMCs) by upregulating the expression of transient receptor potential (TRPC1) (Zhang et al. 2016).

TNF-α and IL-6, highly expressed in the asthmatic subjects (Doganci et al. 2005; Russo and Polosa 2005), are important pro-inflammatory cytokines in regulating asthma pathophysiology. Studies show that single nucleotide polymorphisms (SNPs) of them may be risk factors for asthma susceptibility (Chiang et al. 2013; Daneshmandi et al. 2011; Li et al. 2006). IL-6, an inhibitor of T helper 1 (Th1) differentiation, is a significant modulator of effector CD4+ T cell differentiation and IL-4 production during the process of Th2 differentiation. The ability of regulating Th1 and Th2 differentiation makes IL-6 become a crucial factor in the onset of asthma (Dienz and Rincon 2009; Lee et al. 2017; Neveu et al. 2010).

In our previous unpublished study, we screened differentially expressed lncRNAs in LPS-stimulated P815 mast cells and control P815 mast cells. Subsequent study in vitro indicated that after downregulating the differentially expressed lncRNA-AK149641 in P815 mast cells, the concentrations of TNF-α and IL-6 were significantly reduced in supernatants, suggesting roles of lncRNA-AK149641 in inflammatory response. In this study, microarray analysis also showed that lncRNA-AK149641 expression was twofold higher in OVA mice compared to control mice.

Based on the previous study, in order to further investigate the function and possible mechanism of lncRNA-AK149641 in mediating the airway inflammatory response in vivo, an OVA-induced asthma mouse model was established and studied.

## Materials and methods

### Mice

Specific pathogen-free (SPF) female BALB/c mice, 6–8-week-old, were purchased from the animal core facility of Nanjing Medical University (Nanjing, China), and housed in pathogen-free conditions. All mice were fed with OVA-free food and water for approximately one week before the experiments were started. The study reported here complies with ethical requirements and is permitted by the Animal Ethical and Welfare Committee (AEWC) of Nanjing Medical University (Approval No. IACUC-1703005).

### Establishment of OVA-induced asthma mouse model

Mice were sensitized and challenged as described previously (Li et al. 2015; Suh et al. 2016) with slight modification. That is, BALB/c mice were sensitized at day 0 and day 14 by intraperitoneal (i.p.) injections of the model allergen OVA (V grade) (Sigma-Aldrich, St. Louis, MO, USA) (20 μg OVA, 2 mg Al(OH)_3_ plus phosphate buffered solution (PBS) with a total volume of 0.2 ml). By using an ultrasonic nebulizer (PARI GmbH, Starnberg, Germany), mice were challenged for 20–25 min via the airway by an aerosol consisting of 1% OVA on four successive days (day 27 to day 30). Non-OVA mice received the same schedule for sensitization and challenge with PBS instead of OVA.

### LncRNA microarray assay

Triplicate RNA samples extracted from OVA mice and normal mice were used for lncRNA microarray assays. It was performed by Kangchen Bio-tech Co., Ltd. (Shanghai, China). The thresholds set to identify upregulated or downregulated genes were fold changes of ≥2.0 or ≤ 0.5, respectively.

### AAV6

Recombinant AAV6, with strong affinity for lung tissue and expressed permanently, acted as a vector. It was constructed and packaged by Hanbio Biotechnology Co., Ltd. (Shanghai, China), with a titer over 1 × 10^12^ vg/ml, containing green fluorescent protein (GFP) expression sequence label and sequence of interest. The interest sequence referred to invalid sequence (shRNA-NC) or interference sequence of lncRNA-AK149641 (shRNA-AK149641). ShRNA-AK149641 was used to downregulate the expression of lncRNA-AK149641, while shRNA-NC, without biological significance, was used as control. The sequences of shRNA-NC and shRNA-AK149641 are summarized in Table 1.

**Table 1:** The sequences of shRNA-NC and shRNA-AK149641

	Sequence
shRNA-NC	Top strand: gatccGTTCTCCGAACGTGTCACGTAATTCAAGAGATTACGTGACACGTTCGGAGAATTTTTTc
	Bottom strand: aattcAAAAAATTCTCCGAACGTGTCACGTAATCTCTTGAATTACGTGACACGTTCGGAGAACg
shRNA-AK149641	Top strand: GatccGGTTTGACAGTAGCTAGTTTTCAAGAGAAACTAGCTACTGTCAAACCTTTTTTc
	Bottom strand: aattgAAAAAAGGTTTGACAGTAGCTAGTTTCTCTTGAAAACTAGCTACTGTCAAACCg

### Infection of AAV6

Two days before the first sensitization (day −2), mice were infected with AAV6 to express the interest sequences. After the mice were fully anesthetized (4% chloral hydrate, 0.1 mg/10 g) by i.p. injections, with the help of endotracheal intubation, 50 μl liquid containing AAV6 was infused to the lung.

### Groups

Mice were randomly divided into four groups for different interferences: (1) the control mice (NC): infected by AAV6 containing the sequence of shRNA-NC (AAV6-shRNA-NC), sensitized and challenged by PBS; (2) the mice with lncRNA-AK149641 downregulated (shRNA-AK149641): infected by AAV6 containing the interference sequence of shRNA-AK149641 (AAV6-shRNA-AK149641), sensitized and challenged by PBS; (3) the OVA mice (OVA): infected by AAV6-shRNA-NC, sensitized and challenged by OVA; (4) the OVA mice with lncRNA-AK149641 downregulated (OVA+shRNA-AK149641): infected by AAV6-shRNA-AK149641, sensitized and challenged by OVA. Mice were sacrificed 24 h after the last challenge.

### Total RNA extraction and qRT-PCR analysis

Total RNA was extracted from the lung and other organs by using TRIzol reagent (Invitrogen, Carlsbad, CA, USA), according to the manufacturer’s instructions. One thousand nanograms of total RNA was reverse transcribed in a final volume of 20 μl using a RevertAid First Strand cDNA Synthesis Kit (Thermo Fisher Scientific, Waltham, MA, USA). Quantitative real-time polymerase chain reaction (qRT-PCR) was performed using the FastStart Universal SYBR Green Master (Roche, Switzerland) on an ABI 7500 system (Applied Biosystems, Carlsbad, CA, USA) according to the manufacturer’s instructions. The expression level of lncRNA-AK149641 was normalized to that of GAPDH and calculated using the 2^-ΔΔCT^ method. The sequences of the primers used for lncRNA amplification are summarized in Table 2.

**Table 2:** The sequences of the primers used for lncRNA amplification

	Sequence
AK149641	Forward:5’-GATGCTCTGGAACTGGAGGT-3′
	Reverse: 5’-GCGATGTCTCTGCTGGAAG-3′
GAPDH	Forward:5’-GGTTGTCTCCTGCGACTTCA-3′
	Reverse: 5’-TGGTCCAGGGTTTCTTACTCC-3′

### Measurement of AHR to methacholine

Mice were anesthetized with 1.0% pentobarbital sodium by i.p. injection 24 h after the last challenge, and then AHR was measured by the AniRes2005 Lung Function System (Bestlab, Beijing, China), according to the manufacturer’s instructions and previous study (Li et al. 2013). Airway responsiveness was examined by airway compliance (Cydn) and expiratory airway resistance (R_e_).

### Collection and analysis of bronchoalveolar lavage fluid (BALF)

Airway inflammation was assessed 24 h after the last challenge. BALF was collected by delivering 0.8 ml cold PBS through endotracheal intubation and gently aspirating the fluid. The lavage was repeated three times. The recovery rate was about 80%.

### Histopathological analysis

The lung tissues were obtained to evaluate pathological changes in the lung parenchyma. Samples were fixed in 4% neutral buffered formalin, processed, paraffin embedded, and sectioned at 4-μm thickness. Three different staining methods, that is, hematoxylin and eosin (H&E) staining, periodic acid**-**schiff (PAS) staining, and immunohistochemical staining of TNF-α and IL-6 were used for light microscopic evaluation, as described in previous studies (Ford et al. 2001; Myou et al. 2003). All sample slides for comparison were assessed under the same magnification.

### Western blot analysis

Total protein was separated and transferred onto nitrocellulose membranes (Millipore, MA, USA). Membranes were blocked with 5.0% non-fat milk for two hours at room temperature and incubated overnight at 4°Cwith specific antibodies. After washing with TBST, membranes were incubated with a horseradish peroxidase-conjugated anti-rabbit antibody at room temperature for two hours. Signals were detected on a gel imaging system using an ECL western blotting substrate (Thermo Fisher Scientific, Waltham, MA, USA).

### Statistical analysis

Statistical analysis was performed by using SPSS 20.0 software (SPSS Inc., Chicago, IL, USA). Comparisons of groups were made using Student’s *t* test and multiple comparisons were made using one-way analysis of variance (ANOVA). A difference was considered statistically significant if the *P* value was <0.05.

## Results

### Differentially expressed lncRNAs between the lung tissue of OVA mice and normal control mice

**Fig. 1:**
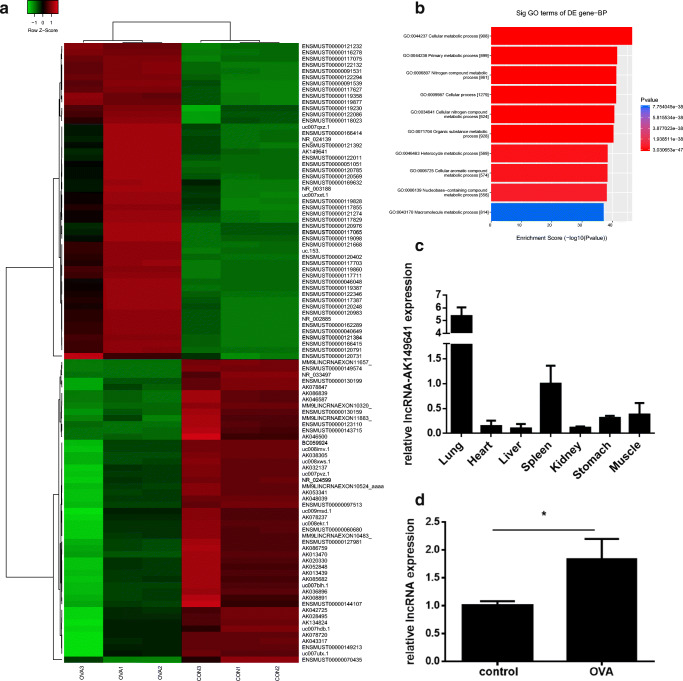
**Result of lncRNA microarray assays and distribution of lncRNA-AK149641 in female BALB/c mice.** (A) The heat map of significantly differentially expressed lncRNAs between OVA mice and control mice. (Mice used for microarray assays were not infected with AAV6; CON1, CON2, and CON3 stood for control mice; OVA1, OVA2 and OVA3 stood for OVA mice). (B) The GO analysis, mainly referred to biological process (BP). (C) Distribution of lncRNA-AK149641 in different organs, qRT-PCR showed that lncRNA-AK149641 expressed the most in the lung tissue. (*n* = 6). (D) LncRNA-AK149641 expressed higher in OVA mice, as determined by qRT-PCR. (*P < 0.05, *n* = 5)

To identify lncRNAs that may be involved in the inflammatory response of asthma, triplicate RNA samples extracted from the lung tissue of OVA mice and control mice were used for lncRNA microarray assays. The heat map of significantly differentially expressed lncRNAs was presented in Fig. 1A. Differentially expressed lncRNAs were classified into five categories: intergenic, exon sense-overlapping, intronic antisense, natural antisense, and bidirectional lncRNAs. Bioinformatics analysis such as gene ontology (GO) was performed (Fig. 1B). The bar plot showed the top ten of the fold enrichment value of the significant enrichment terms. LncRNA microarray analysis showed that lncRNA-AK149641 expressed differentially in OVA mice (fold change: 4.52), consistent with our previous unpublished study in vitro. To further investigate the distribution of lncRNA-AK149641 in vivo, different organs from female BALB/c mice were obtained. QRT-PCR revealed that expression of lncRNA-AK149641 in the lung tissue was four to five times higher than that in other organs, as shown in Fig.1C**.** Moreover, we also found that in OVA mice, lncRNA-AK149641 upregulated significantly (Fig.1D). Therefore, we focused on lncRNA-AK149641 in the subsequent study.

### Establishment of mouse model

**Fig. 2:**
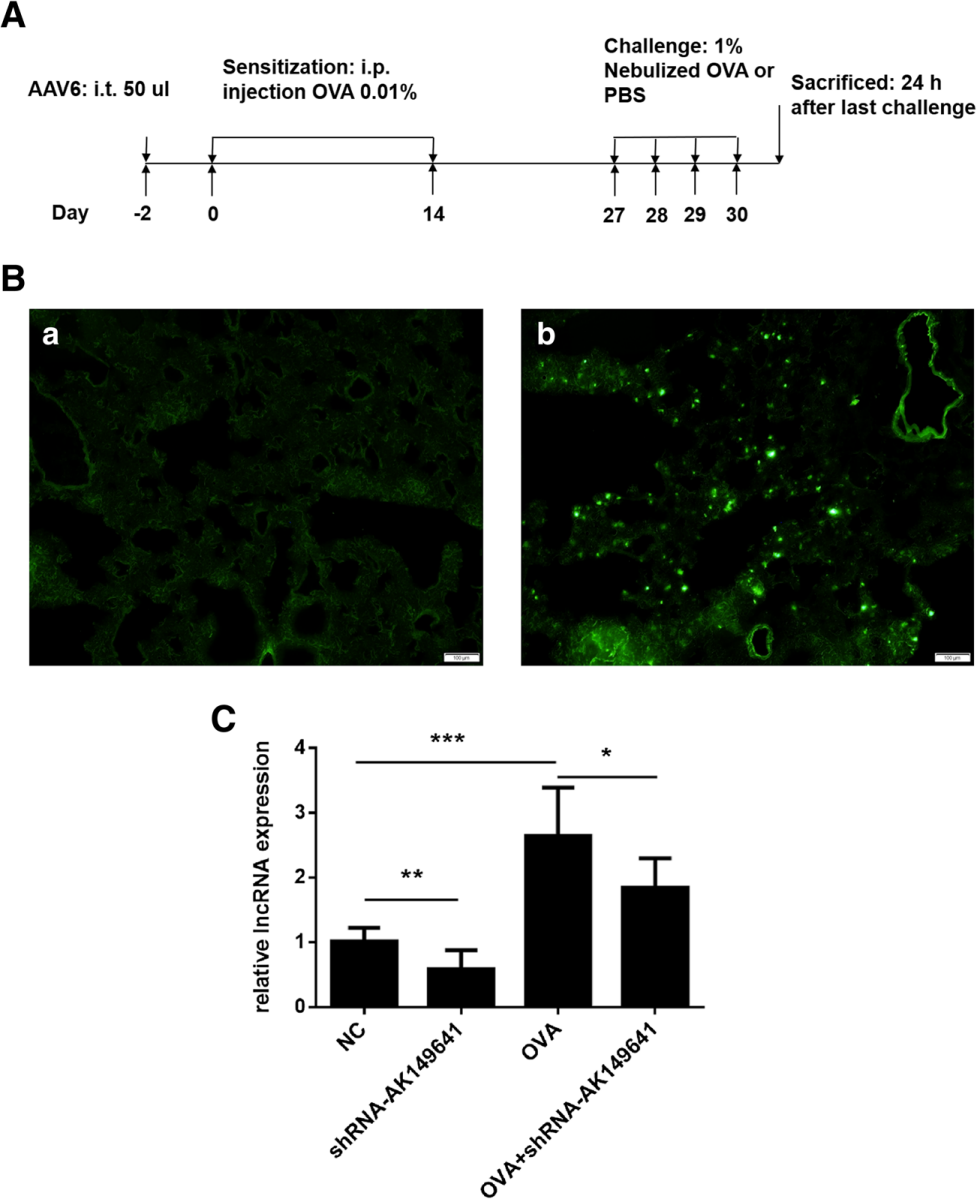
**AAV6 infected the lung tissue and AAV6-shRNA-AK149641 downregulated the expression of lncRNA-AK149641 successfully.** (A) Brief protocol for the establishment of OVA-induced asthma mouse model. (B) GFP was observed in the lung tissue of mice which infected with AAV6 (b), while it was negative in the mice without AAV6 infection (a), suggesting that AAV6 infected the lung tissue successfully. (C) Different expressions of lncRNA-AK149641 in the lung tissue with different interventions, as determined by qRT-PCR. The expression of lncRNA-AK149641 expressed higher in the OVA mice. Infected with AAV6-shRNA-AK149641 showed lower expression, with statistical difference, suggesting that AAV6-shRNA-AK149641 could successfully down-regulate the expression of lncRNA-AK149641. (*P < 0.05, **P < 0.01, ***P < 0.001, *n* = 6)

Protocol of establishment of OVA-induced asthma mouse model was showed in Fig. 2A. Twenty-four hours after last challenge, lung tissues and BALF were obtained, and AHR was measured.

### Infection of AAV6-shRNA-AK149641 decreased the expression of lncRNA-AK149641 in the lung tissue

Analysis of frozen lung tissue slices showed that AAV6 successfully infected the lung tissue, as GFP was observed by using fluorescence microscopy (Fig. 2B). As shown in Fig. 2C, lncRNA-AK149641 expressed higher in OVA mice compared with non-OVA mice. On the other hand, when infected with AAV6-shRNA-AK149641, expression of lncRNA-AK149641 was reduced, with statistical significance, suggesting that AAV6-shRNA-AK149641 significantly downregulated lncRNA-AK149641 expression in the lung tissue.

### Downregulation of lncRNA-AK149641 attenuated AHR in OVA mice

**Fig. 3:**
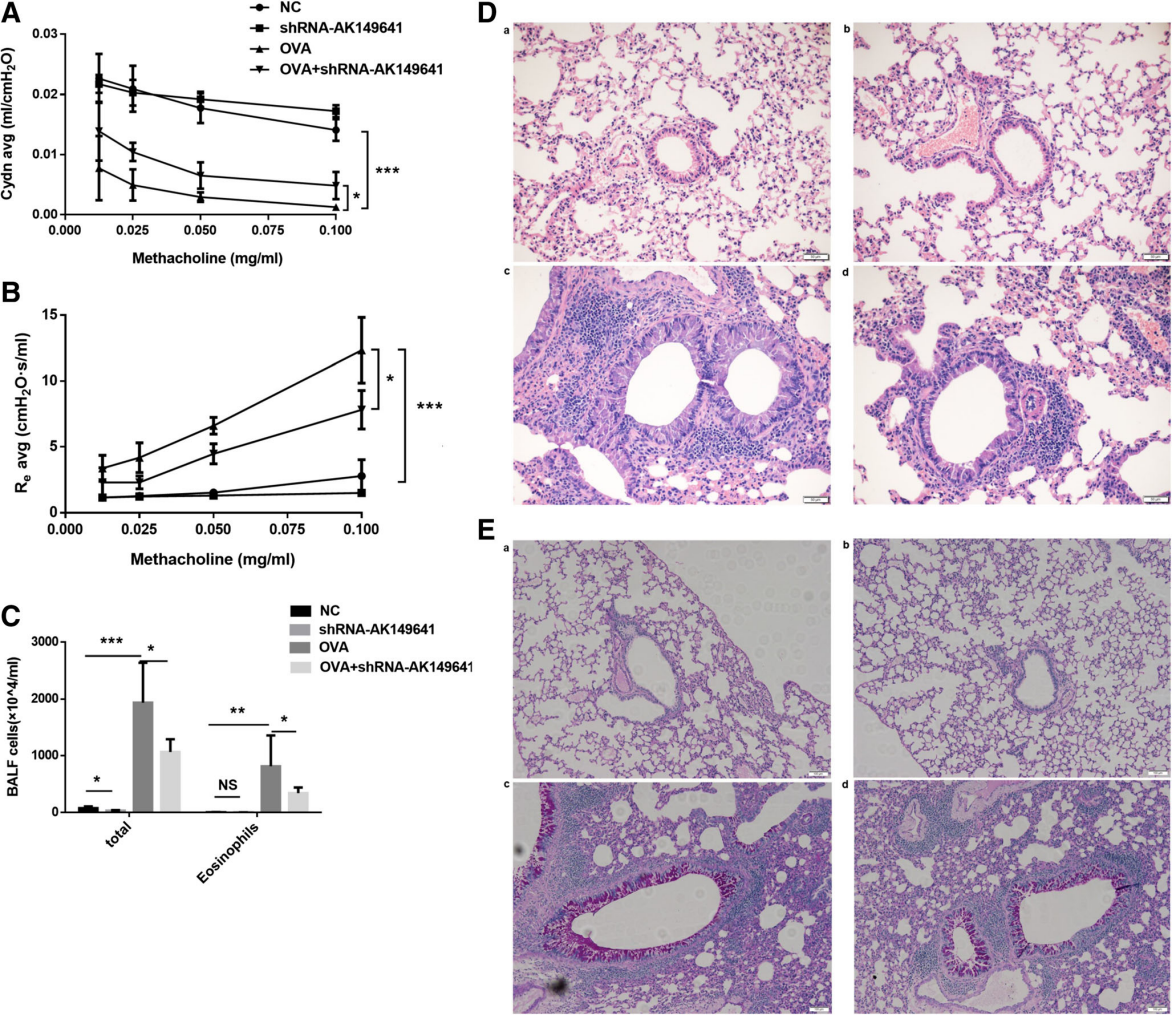

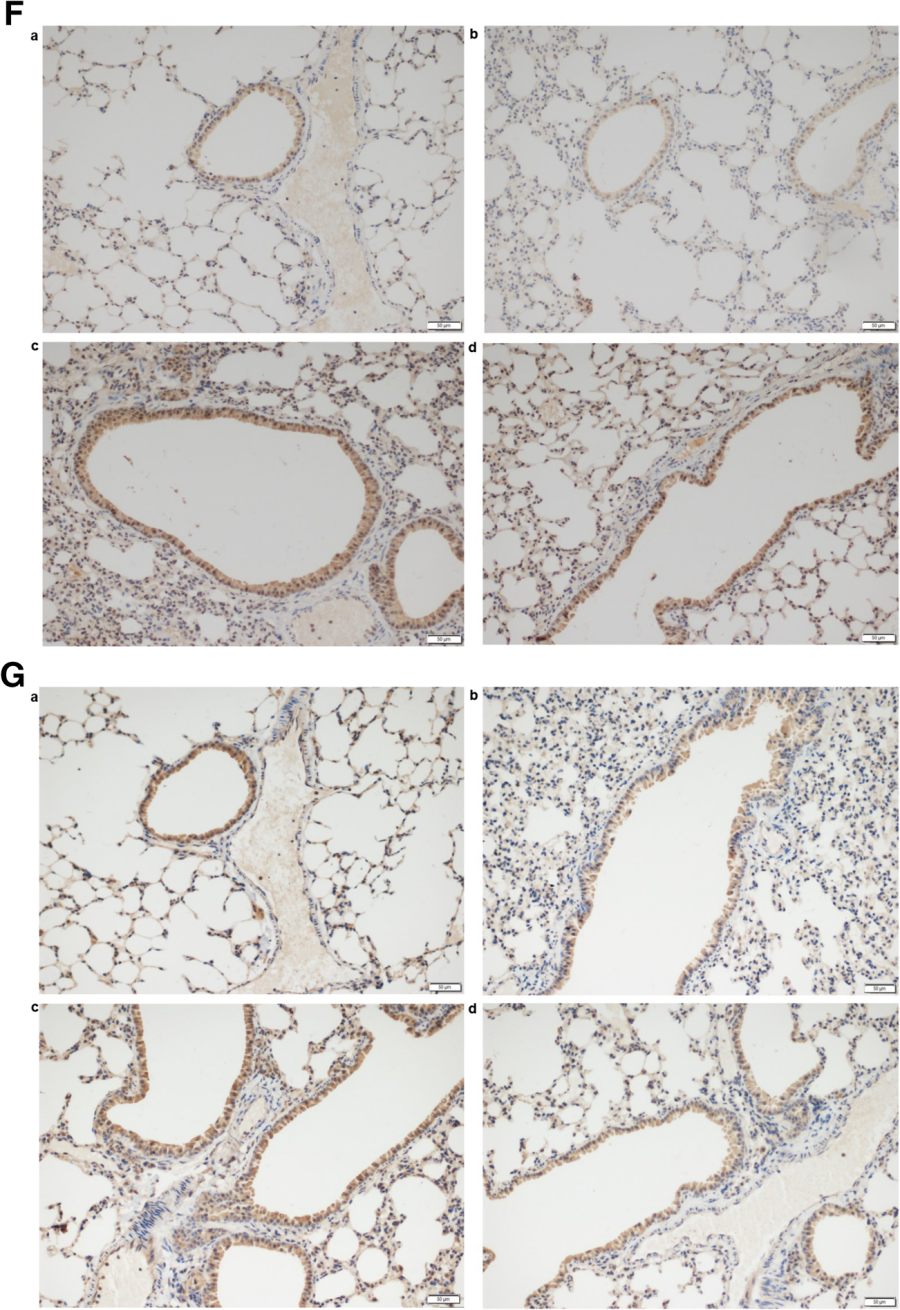
**Downregulation of lncRNA-AK149641 attenuated the airway inflammatory response in OVA mice.** (A) Airway compliance (Cydn) decreased and (B) airway resistance (R_e_) increased in the OVA mice, while downregulating lncRNA-AK149641 weakened the changes, when the concentration of methacholine was over 0.0125 mg/ml. (**P < 0.05, *n* = 4). (C) Total inflammatory cells and eosinophils infiltrated higher in BALF of OVA mice, while downregulating lncRNA-AK149641 reduced the infiltrations. All the differences were statistically significant. (*P < 0.05, **P < 0.01, ***P < 0.001; *n* = 6). (D) H&E staining of the lung tissue. Downregulation of lncRNA-AK149641 mitigated the peribronchial infiltration of inflammatory cells in OVA mice. (E) PAS staining of the lung tissue. Downregulation of lncRNA-AK149641 mitigated the production of mucus in OVA mice. (F) Immunohistochemical staining of TNF-α and (G) IL-6 in lung tissue. Downregulation of lncRNA-AK149641 decreased expression of TNF-α and IL-6 in OVA mice. (a. NC, b. shRNA-AK149641, c. OVA, d. OVA+shRNA-AK149641). (*n* = 6)

Twenty-four hours after the last challenge, AHR was measured following methacholine (Mch) challenge. As shown in Fig. 3A, B, increasing concentrations of Mch significantly decreased airway compliance (Cydn) and increased asthmatic expiratory airway resistance (R_e_) in OVA mice. However, when lncRNA-AK149641 was downregulated in OVA mice, these changes were inhibited. Of note, downregulation of lncRNA-AK149641 had no effect on non-OVA mice in aspect of AHR. Taken together, downregulation of lncRNA-AK149641 showed a benefit on AHR in OVA mice.

### Downregulation of lncRNA-AK149641 significantly reduced total cell counts and eosinophil counts in BALF of OVA mice

To identify whether lncRNA-AK149641 could affect the infiltration of inflammatory cells in OVA mice, BALF samples were obtained and examined. As shown in Fig. 3C, OVA mice exhibited robust increases in total cell counts and eosinophil counts, while downregulating lncRNA-AK149641 significantly decreased the infiltration of inflammatory cells. In contrast, non-OVA mice exhibited minimal inflammatory cell numbers in BALF and lower expression of lncRNA-AK149641 had no effect on eosinophil counts. Thus, downregulation of lncRNA-AK149641 attenuated asthmatic airway inflammation by reducing infiltration of inflammatory cells.

### Downregulation of lncRNA-AK149641 reduced infiltration of inflammatory cells and production of mucus in the lung tissue of OVA mice

Microscopically, exposure to OVA markedly increased peribronchial infiltration of inflammatory cells compared with non-OVA mice, and downregulation of lncRNA-AK149641 mitigated the infiltration, as shown by H&E staining (Fig. 3D), which was consistent with the result obtained in the BALF analysis. With low expression of lncRNA-AK149641 in OVA mice, PAS staining of the lung tissue showed that mucus production was also dramatically decreased (Fig. 3E). Based on histological evaluation, we concluded that downregulation of lncRNA-AK149641 mitigated the peribronchial infiltration of inflammatory cells and production of mucus.

### Downregulation of lncRNA-AK149641 decreased TNF-α and IL-6 in OVA mice

Concentrations of TNF-α and IL-6 in the lung tissue were assessed by immunohistochemistry to determine whether lncRNA-AK149641 affected their expressions. Both inflammatory factors were highly expressed in OVA mice. However, infection of AAV6-shRNA-AK149641 resulted in significantly lower expression levels of TNF-α and IL-6 (Fig. 3F, G).

### Activity of NF-κB signaling pathway decreased in lncRNA-AK149641 downregulated mice

**Fig. 4:**
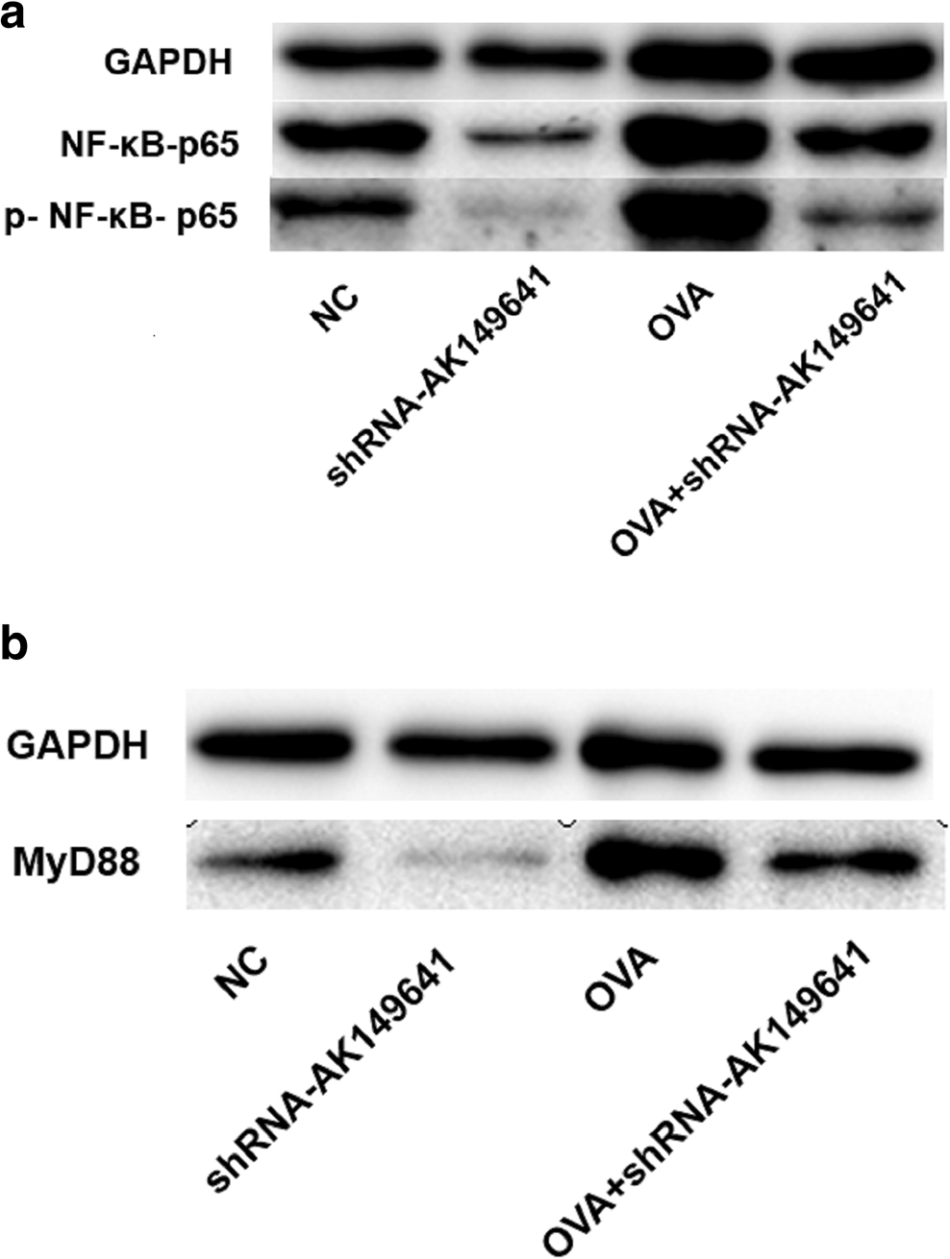
**Changes in protein expression of NF-κB signaling pathway components in response to different lncRNA-AK149641 levels.** (A) p-NF-κB p65 and (B) MyD88 protein levels in lung tissue, as determined by western blot. (repeated three times)

As shown in Fig. 4A, B, compared to non-OVA mice, expressions of phosphorylated NF-κB p65 (p-NF-κB p65) and MyD88, as determined by western blot, were increased in OVA mice, whereas downregulation of lncRNA-AK149641 significantly decreased their expression levels. In summary, we determined that lncRNA-AK149641 participated in the asthma-associated inflammatory response via the NF-κB signaling pathway probably by regulating expressions of p-NF-κB p65 and MyD88.

## Discussion

Microarray analysis revealed that there were differentially expressed lncRNAs between the lung tissue of OVA mice and control mice. Another study (Zhu et al. 2018) found 41 dysregulated lncRNAs (difference ≥ 2-fold) in blood samples from patients with eosinophilic asthma, compared to the healthy individuals’, supporting that lncRNAs were related to asthma. Deeper researches focused on the mechanism on how lncRNAs regulated asthma. Lin et al. proposed that lncRNA TUG1 promoted ASMCs proliferation and migration via sponging miR-590-5p/FGF1 in asthma (Lin et al. 2019a). LncRNA TCF7 was found to be contributed to the growth and migration of ASMCs in asthma through targeting TIMMDC1/Akt axis (Fan et al. 2019).

TNF-α and IL-6 are major pro-inflammatory cytokines involved in asthma. In asthma mouse model, anti-TNF-α treatment attenuated airway inflammation (Kim et al. 2006). Moreover, anti-TNF therapy resulted in clinical improvement of AHR, lung function and quality-of-life of patients with asthma, together with a reduction in exacerbation frequency (Berry et al. 2007). IL-6 is related to asthma severity. Sputum IL-6 level was inversely related to FEV1 (% predicted) and positively correlated with the Asthma Control Questionnaire (ACQ) score (Morjaria et al. 2010).

LncRNAs and pro-inflammatory cytokines are closely associated with inflammation. Our results revealed that expressions of TNF-α and IL-6 were lower in OVA mice upon downregulating lncRNA-AK149641, suggesting that lncRNAs and cytokines may cooperate to modulate inflammation. In human abdominal aortic aneurysm, IL-6 induced the activity of NOX2 in the aortic endothelial cells via the induction of the lncRNA MALAT1 by an ERK-dependent mechanism (Wang et al. 2016). The lincRNA THRIL, whose expression levels correlated with the severity of Kawasaki disease, induced expression of TNF-α by forming a ribonucleoprotein (RNP) complex with hnRNPL (Li et al. 2014).

PAS staining showed that secretion of mucus decreased in lncRNA-AK149641 downregulated OVA mice, suggesting that lncRNA-AK149641 maybe a regulator of mucus production. A recent study also revealed that linc00632 inhibited IL-13-induced muac5ac production in nasal epithelial cells (Yue et al. 2020).

LncRNAs and different cell types were involved in the pathophysiology of asthma. Studies showed that increased lncRNA H19 inhibited the function of goblet cells, thus potentially contributing to barrier dysfunction in intestinal pathologies (Yu et al. 2020). H19 was found differentially expressed in OVA mice in our microarray analysis, thus, H19 could also potentially regulate inflammation of asthma by targeting goblet cells. LncRNA-AK085865 depletion can ameliorate the inflammation of dermatophagoides farinae protein 1-induced allergic asthma by modulation macrophage polarization (Pei et al. 2020). In human patients with asthma, lncRNA-MEG3 regulated Treg/Th17 balance (Qiu et al. 2019). Many reports revealed that lncRNAs regulated asthmatic inflammation through ASMCs. Upregulation of MALAT1 took part in platelet-derived growth factor (PDGF)-BB-induced ASMC proliferation and migration (Lin et al. 2019b). LINC00882 contributes to PDGF-induced proliferation of human fetal ASMCs by enhancing Wnt/β-catenin signaling via miR-3619-5p. Glucocorticoid is an important treatment for asthma, while lncRNA GAS5 is a decoy for glucocorticoid receptor. In asthmatic rats, GAS5 promotes ASMC proliferation by miR-10a/BDNF signaling pathway (Zhang et al. 2018). Christine et.al. found that proinflammatory mediators up-regulate GAS5 levels in both airway epithelial and smooth muscle cell, and that decreasing GAS5 levels can enhance glucocorticoid action in airway epithelial cells.

The transcription factor NF-κB is an essential regulator in inflammation, immunity, differentiation, and cell proliferation (Ruland 2011). The NF-κB family consists of five subunits: p50, p52, p65 (RelA), c-Rel, and RelB. Among which, p65 (RelA), c-Rel, and RelB obtain transcription activation domain and usually activate gene transcription. Recent studies have shown that lncRNAs take part in pathophysiological process by regulating NF-κB signaling pathway. Wang et al. reported that lncRNA BANCR regulated NF-κB1 (P50/105) via miR-9 to influence cell growth and apoptosis in gastric cancer (Zhang et al. 2015). LPS induced lncRNA, Mirt2, specifically inhibited the K63-ubiquitination of TRAF6, and thus, inhibiting the activation of NF-κB and MAPK pathways to limit production of proinflammatory cytokines (Du et al. 2017). LncRNA NKILA inhibited IKK-induced IκB phosphorylation and NF-κB activation by forming a stable complex with NF-κB/IκB, thus preventing over-activation of NF-κB pathway to suppress breast cancer metastasis (Liu et al. 2015).

Downregulation of lncRNA-AK149641 in OVA mice resulted in significant decrease in the protein levels of p-NF-κB p65, providing a potential possibility that lncRNA-AK149641 regulates NF-κB, probably through p65 subunit. In endothelial cells, TNF-α induced the expression of lncRNA ANRIL through NF-κB/p65. When bound to the transcriptional factor YY1, ANRIL regulated expressions of IL-6 and IL-8, downstream of NF-κB signaling pathway (Zhou et al. 2015b). In mouse tubular epithelial cells, lncRNA Arid2-IR played the promoter role in NF-κ B-dependent renal inflammation by infecting IL-1β-induced phosphorylation and DNA binding activity of NFκB/p65 (Zhou et al. 2015a). As for our study, to better understand how lncRNA-AK149641 regulates NF-κB signaling pathway, electrophoretic mobility shift assays (EMSA) and RNA binding protein immunoprecipitaiton (RIP) will be done in the future.

In summary, downregulation of lncRNA-AK149641 in OVA mice attenuated AHR, decreased infiltrations of inflammatory cells in BALF and peribronchia, reduced secretions of TNF-α, IL-6 and glycogen, in association with decreased expression of MyD88, and p-NF-κB p65. We propose that lncRNA-AK149641 regulates the asthma-associated airway inflammatory response by targeting the NF-κB signaling pathway.
